# Multiscale correlations between joint and tissue-specific biomechanics and anatomy in postmortem ovine stifles

**DOI:** 10.1038/s41598-025-87491-w

**Published:** 2025-02-07

**Authors:** Aritra Chatterjee, Zachary Robert Davis, Timothy Lescun, Deva D. Chan

**Affiliations:** 1 Weldon School of Biomedical Engineering, Purdue University, 206 S. Martin Jischke Drive, West Lafayette, IN 47907 USA; 2 Department of Mechanical Engineering, Birla Institute of Technology & Science, Pilani, Hyderabad Campus, Hyderabad, Telangana 500078 India; 3 Department of Veterinary Clinical Sciences, College of Veterinary Medicine, Purdue University, West Lafayette, IN 47907 USA; 4 School of Mechanical Engineering, Purdue University, West Lafayette, IN 47907 USA

**Keywords:** Joint laxity, Tissue mechanics, MRI, Viscoelasticity, *T*_2_^*^ relaxation time, Biomedical engineering, Musculoskeletal system

## Abstract

Joint stability depends on various properties that include the bone anatomy, joint capsule geometry and stiffness, and soft tissues within. The multiscale biomechanical relationships between the whole joint and individual tissues are useful in estimating the physiological condition of the knee. To better understand these relationships, we evaluated multiple structural and mechanical parameters in healthy ovine stifles, specifically joint laxity, joint morphology, individual tissue *T*_2_^*^ relaxation and mechanical properties of ligaments (ACL, PCL, MCL, LCL), patellar tendon, menisci, and cartilage. By combining mechanical testing at two length scales and magnetic resonance imaging (MRI) scans, we quantify the strength of correlation coefficients between measured metrics, among joint size, whole joint and individual tissue properties. We observe positive correlations between joint laxity forces and the inter-epicondylar distance. Further, the viscoelastic properties of the tendons and ligaments correlate positively with joint laxity forces; however, no such correlations were observed for the cartilage and menisci. We also found weak inverse correlation between tissue viscoelasticity and *T*_2_^*^ for MCL; and positive correlations for cartilage and menisci, LCL and PCL respectively. These results provide useful insights into the role of individual tissues that are crucial in measuring whole joint responses as key indicators of knee health.

**Supplementary Information:**

The online version contains supplementary material available at 10.1038/s41598-025-87491-w.

## Introduction

The knee is subject to a wide range of complex loading conditions that depend on multiple factors such as the body weight of the individual, the frequency and extent of movement of the limbs, and their energy absorption capacity^1^. Despite its importance in everyday mobility and quality of life, the knee is susceptible to different types of injuries, affecting nearly 46% adults over their lifetime^2^. Injuries to or impairment of the knee can significantly impact human mobility and well-being^3^. The anatomic structures within the knee, the connective tissues including the tendons, ligaments together with the meniscus and articular cartilage all contribute significantly influencing the movement and stability of the joint^4^. A thorough understanding of the structure and functional properties of the knee joint, and its underlying structures are required to identify the cause of such injuries and develop better diagnostic or precautionary measures^5^. Multiscale biomechanical studies of the knee joint in large animal models that can closely capture the human joint anatomy and loading conditions, are often useful in designing such studies. Owing to their low-cost relative to other large animals, and physiological similarities with humans, ovine models are commonly used in orthopedic research and late-stage preclinical studies^6^.

Joint laxity, often widely used as a metric to quantify the stability of the joint, is defined as a measurement of the net joint movement under the application of an external force during a state of muscular relaxation^7^, and is linked to joint instability which can arise because of injuries to soft tissues like ligament tear or in case of degenerative diseases like osteoarthritis^8^. Anterior drawer tests are a routinely used technique to quantify joint laxity in a clinical setting^9,10^ and provide advantages over whole joint compression tests, which may cause damage to the soft tissues under high loads^11^ and can subsequently impact the MRI scans and tissue mechanical tests. Laxity depends on multiple factors including the mechanical constraints of the underlying soft tissues, tibio-femoral bone shape^12^, along with the biomechanical properties of the cartilage and meniscus^13,14^. Investigating the specific correlations between the individual tissue mechanics and structure with joint laxity can help us understand the relationship between the macroscale joint mechanics and the aggregate mechanical properties of the soft tissues.

Magnetic resonance imaging (MRI) is a widely used technique to image knee anatomy^15^, and quantitative relaxometry is useful to noninvasively estimate the structural and material properties of soft tissues, as it is sensitive to changes in water content, proteoglycans, and collagen fibre orientation^16–18^. Both transverse relaxation time (*T*_*2*_) and effective *T*_*2*_ (*T*_2_^*^) have been shown to correlate to cartilage degeneration^19^. An increase in the *T*_*2*_ correlates with damage to articular cartilage, signifying deterioration of the collagen network and increase in the water content^20^. *T*_*2*_ relaxometry have also been previously shown to correlate with the structural and material properties of the ligaments, like the anterior cruciate ligament (ACL)^21^ and posterior cruciate ligament (PCL)^22^, and tendon^23^ in humans. As *T*_*2*_-MRI measurements can quantify the macromolecular composition, collagen fiber orientation, and water content of the soft tissues^24,25^, these measurements can be also be correlated with the mechanical properties of the tissues, such as stiffness^26^ and used to generate subject-specific models that account for regional differences in material properties^27^. Understanding the relationships between soft tissue biomechanics measured ex vivo and *T*_2_^*^ values in healthy tissues can provide the necessary baseline data that can be further extended to computational modelling of healthy joints and studying diseased conditions like osteoarthritis. To better understand these relationships at whole joint and tissue level, in this study, we have performed tissue specific *T*_2_^*^ mapping for the ligaments (ACL, PCL, MCL, PCL), the patellar tendon, the medial and lateral menisci and articular cartilage to determine how well overall joint and individual tissue mechanics correlate with *T*_2_^*^ values.

The passive mechanical responses of the entire knee joint is governed by the mechanical properties of the four ligaments (medial and lateral collateral ligaments (MCL, LCL), ACL and PCL^4^, the patellar tendon^28^, the medial and lateral menisci and articular cartilage^29,30^. The tendons and ligaments mainly act under tensile loads while the meniscus and cartilage primarily support compression along with undergoing hoop stresses, shear and transverse loads during movement. While there have been certain theoretical approaches like the lumped parameter models to simulate the knee joint as an ensemble of the underlying tissues, these models face certain restrictions, in incorporating the tissue geometry and their nonlinear elastic properties^31^. A majority of the current multiscale computational models are based on tissue properties sampled from different studies comprising of different animal models and individuals, which reduce their reliability due to variability in sampling, specimen size and condition, and variation in measurement techniques^32,33^. In a previous report by Peters, et al.^34^, , it has been shown that most of the current finite element models suffer a limitation due to high variability in sample age, species, or the specific locations from which the tissue material properties are tested, thereby reducing their efficacy. As tissue material properties vary with age, sex, and disease status^35^, it is important to develop a more subject-specific approach to develop computational models that can quantitatively predict changes in the structural and mechanical responses of the knee joint, under healthy aging or diseases like osteoarthritis, where all the material properties, loading conditions and the imaging inputs are derived from the same specimen using consistent test conditions, underlying a major motivation of our study. However, to our current knowledge, no studies adopt a multiscale, experimental approach to evaluate the stifle or knee biomechanics by determining the individual structural and functional properties of the underlying soft tissues and laxity properties of the same joint.

**Fig. 1:**
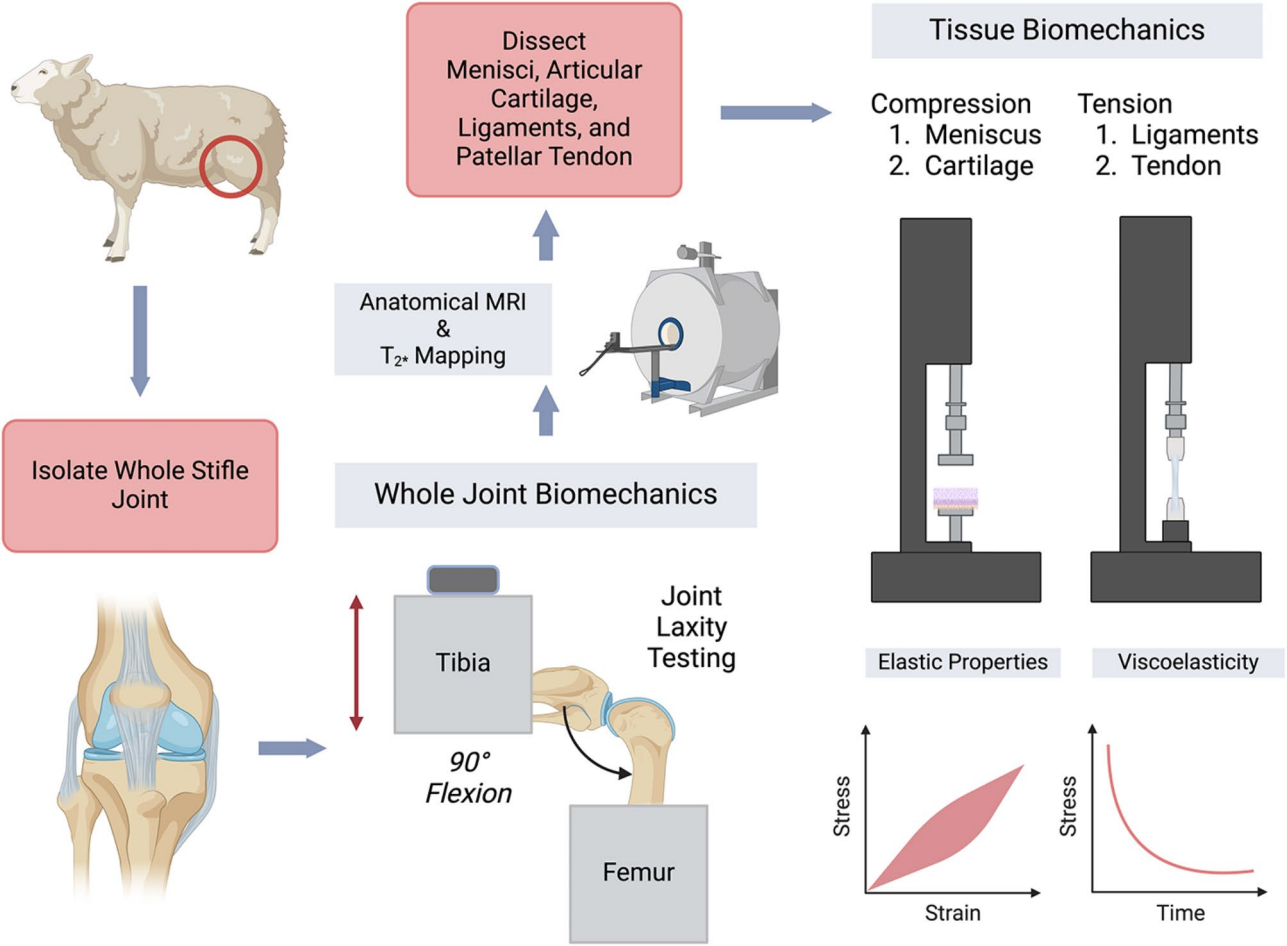
A schematic showing the experimental workflow for multiscale biomechanical testing and imaging of post-mortem ovine stifles. After collection of the ovine stifles (location indicated on sheep with red circle), the excess musculature was removed, keeping the joint capsule intact and the samples were prepared for joint-laxity testing. Following laxity testing, the stifles were imaged in MRI to quantify joint anatomy and *T*_2_^*^ relaxation maps for tissues including the cartilage, menisci, ligaments, and patellar tendon. After imaging, the stifles were dissected, and the individual tissues were extracted in specific geometries suitable for mechanical testing. Cartilage and menisci samples were tested under compression while tendons and ligaments were tested under tensile loading. The corresponding data were used to quantify the elastic and viscoelastic properties of the tissue explants. Schematic created with Biorender.com.

For a deeper understanding of some of these open questions, in this study, we investigate the relationships between whole joint and individual tissue properties using a combination of mechanical testing and imaging techniques. The principal objective of our study is to employ a multiscale approach to quantify the correlations between the whole joint biomechanics and joint size, tissue material properties, and *T*_2_^*^ values of soft tissues within the stifle (Fig. 1). Specifically, we performed joint laxity experiments to compare the results at a whole joint level, with parameters from joint morphology using anatomical MRI scans, individual tissue structure and mechanical properties, collected from the anterior and posterior cruciate ligaments (ACL, PCL), medial and lateral collateral ligaments (MCL, LCL), the patellar tendon, menisci, and femoral cartilage from six different ovine specimens obtained using tissue-specific mechanical testing and quantitative MRI scans to measure *T*_2_^*^ values. We combine mechanical testing at two length scales with anatomical and quantitative MRI scans, to investigate whether there are any significant correlations between the individual tissue and the whole joint properties.

Together, our results reveal relationships between whole joint mechanics, joint size, and individual tissue properties. We show positive correlations between the joint laxity forces and the inter-epicondylar distance for the tested ovine specimens and observe direct correlations with the ligament and tendon tissue mechanics. These results can provide insights into the differential role of individual tissue properties to the overall joint responses, which can help with designing subject-specific multiscale computational models to simulate joint biomechanics more accurately. From a clinical perspective, the framework of these studies can be extended in future to humans to develop improved patient-specific treatment strategies for improved joint health care.

## Results

### Joint laxity measurements correlate with ovine stifle size

**Fig. 2:**
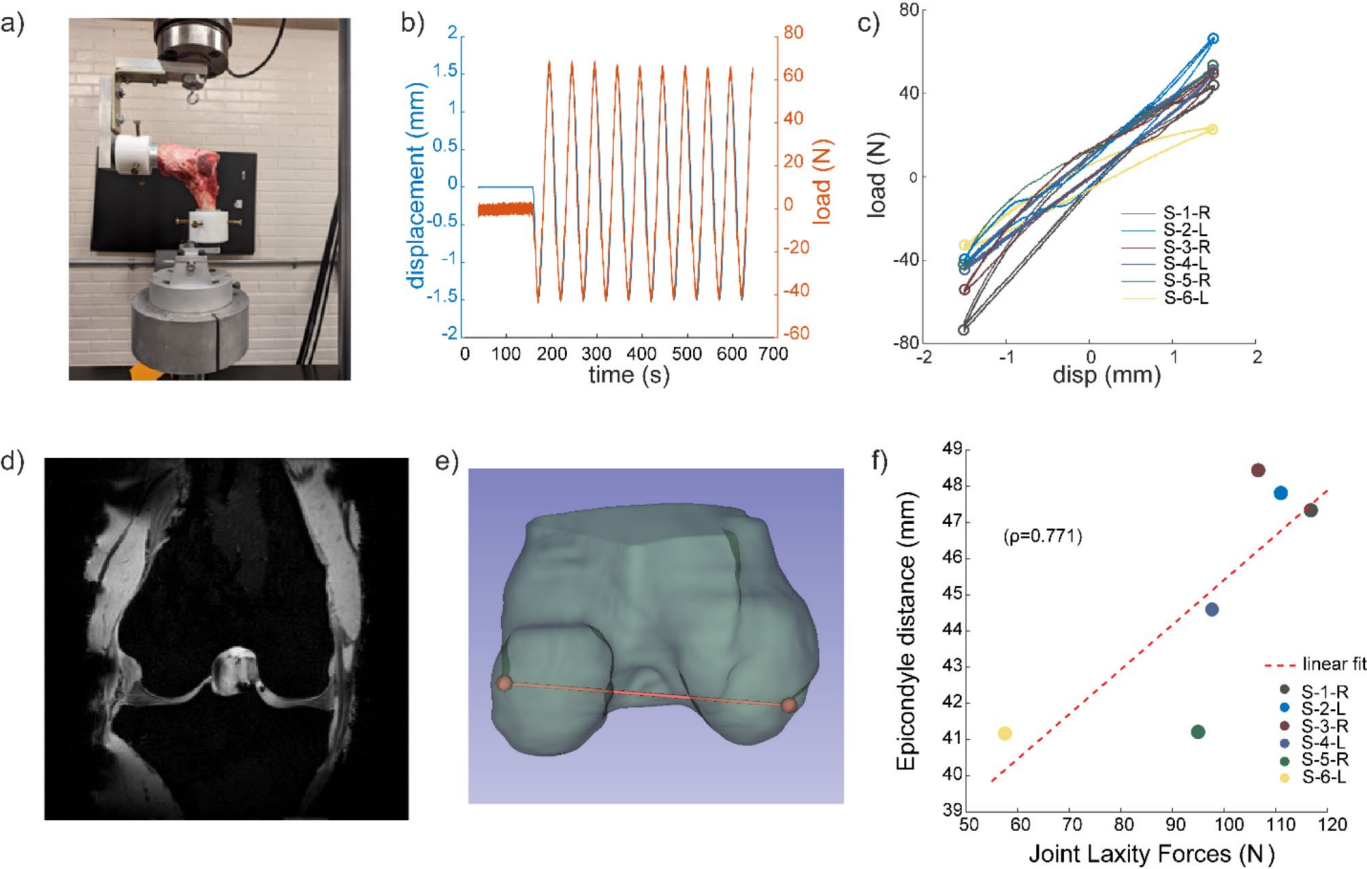
Measurement of Joint Laxity forces and its correlations with stifle size. (a) Ovine stifles were clamped at 90° flexion to a 10-kN load frame for joint laxity testing. (b) Cyclic load (N) and displacement (mm) profiles over time obtained during laxity test. (c) Corresponding load vs. displacements relationships were quantified during joint laxity tests for 6 ovine stifles. (d) 3D *T*_*1*_-weighted FLASH images (representative image from specimen S1-R) were used to segment the femur. (e) Epicondyle-to-epicondyle distance was measured from the segmented femur using 3D Slicer software. (f) Joint laxity forces positively correlated with epicondylar distance.

Laxity testing of the ovine stifles (Fig. 2) under ten ± 1.5-mm displacement cycles resulted in a 90.25 ± 32.33 N load, measured as the difference between the peak forces at the maximum and minimum displacements, across all six ovine specimens (Fig. 2c). We also observed that the magnitude of peak forces in the anterior and posterior direction measured for each specimen differed (Fig. 2c, Supplemental Table 1). The femoral epicondyle-to-epicondyle distance positively correlated (ρ = 0.771, *p* = 0.103) to joint laxity forces measured from the laxity experiments (Fig. 2f). We also quantified the correlation between joint laxity forces and epicondylar distance for the four female specimens and similarly observed a strong positive correlation (ρ = 0.89, *p* = 0.033).

### *T*_2_^*^ relaxation time does not significantly correlate to joint laxity forces

**Fig. 3:**
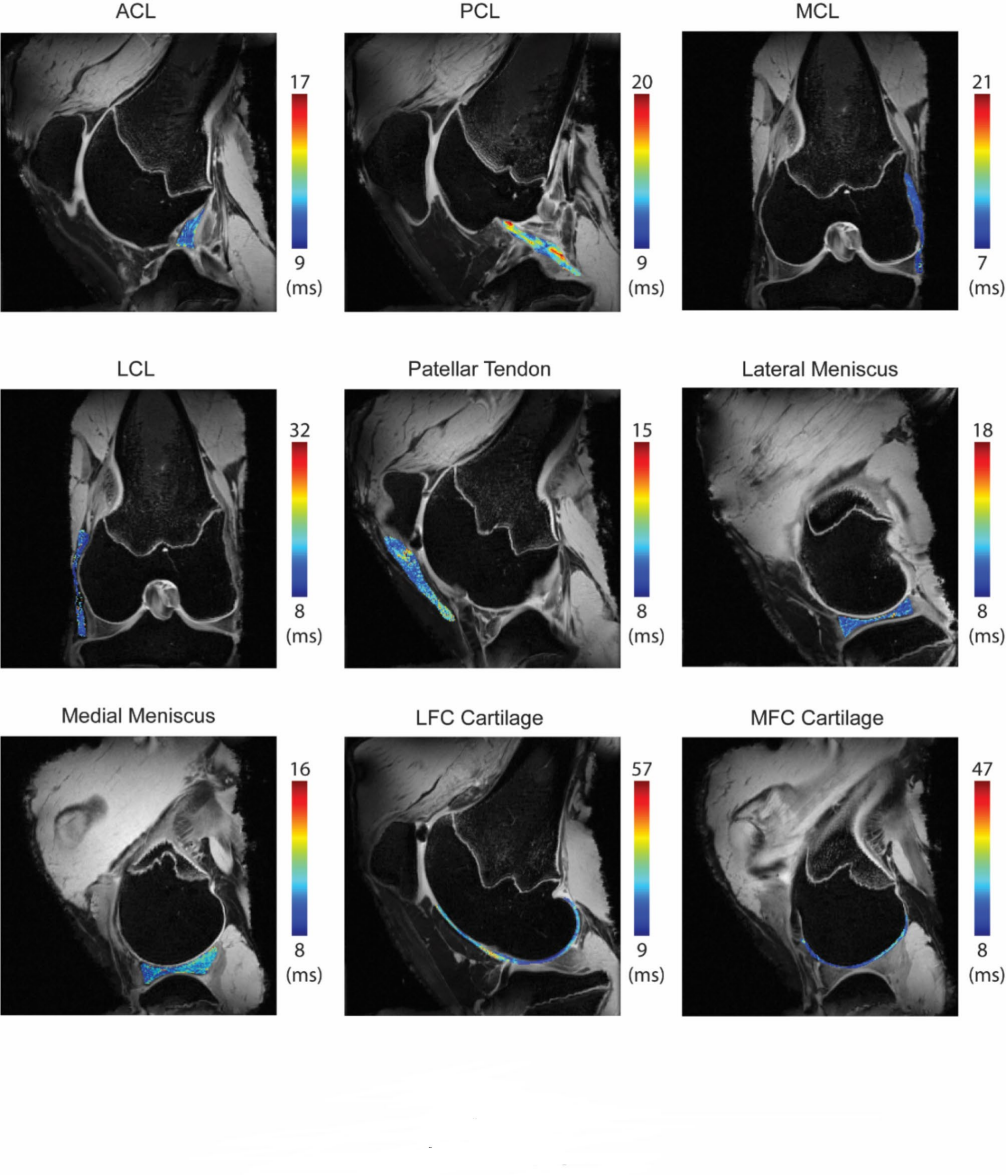
*T*_2_^*^ maps were calculated from 2D multi-slice *T*_2_^*^ multi gradient echo sequence and shown in representative slices for each tissue. Slices that show the *T*_2_^*^ map for the anterior cruciate ligament (ACL), posterior cruciate ligament (PCL), medial collateral ligament (MCL), lateral collateral ligament (LCL), patellar tendon, lateral and medial menisci, and cartilage from the lateral femoral condyle (LFC) and media femoral condyle (MFC) are shown for a representative stifle (animal aged 25 months).

**Fig. 4:**
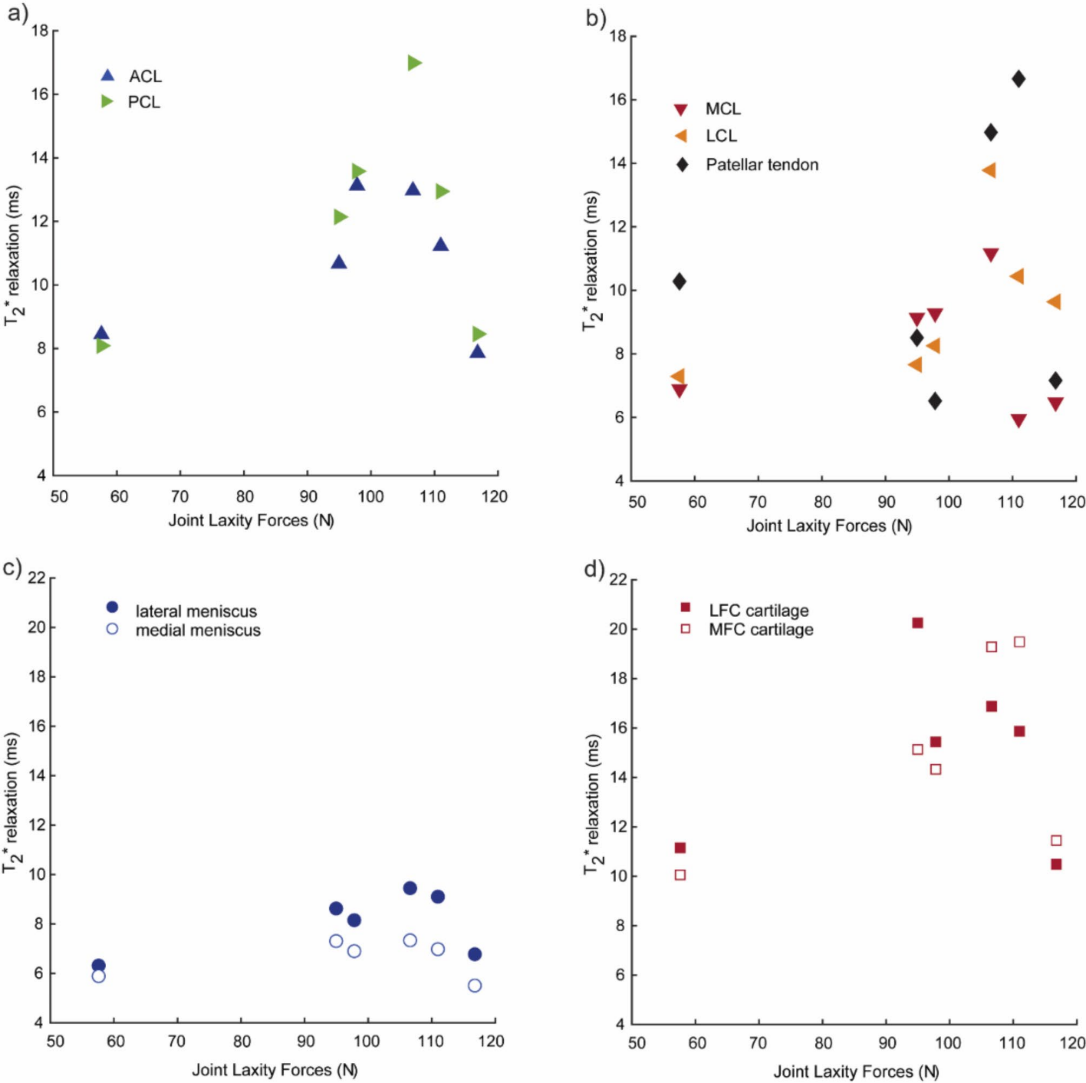
Joint laxity forces (N) are compared with the measured mean *T*_2_^*^ relaxation values (ms) for the different set of tissues. Joint laxity forces and *T*_2_^*^ relaxation time were evaluated for (a) ACL and PCL, (b) the patellar tendon, MCL and LCL, (c) lateral and medial menisci and d) cartilage from lateral and medial femoral condyles.

*T*_2_^*^ relaxation times were calculated for the stifle ligaments (ACL, PCL, MCL and LCL), cartilage, meniscus, and patellar tendon (Fig. 3) and are reported as mean ± standard deviation of for each of the six ovine specimens (Supplemental Table 2), with corresponding R^2^ for goodness of *T*_2_^*^ fit (Supplemental Fig. 1). Within each tissue type, no statistically significant differences were observed in the mean *T*_2_^*^ values among the six different specimens. Some weak and moderate positive correlations were observed between the joint laxity forces and tissue-specific *T*_2_^*^ relaxation times for some tissues (Fig. 4, Supplemental Table 3): LCL (ρ = 0.77, *p* = 0.103), cartilage from the lateral femoral condyles (ρ = 0.37, *p* = 0.49), and lateral menisci (ρ = 0.31, *p* = 0.56); and a weak negative correlation for MCL (ρ=-0.37, *p* = 0.49). However, none of these reached statistical significance.

### Joint laxity forces positively correlate with tendon and ligament viscoelastic properties

**Fig. 5:**
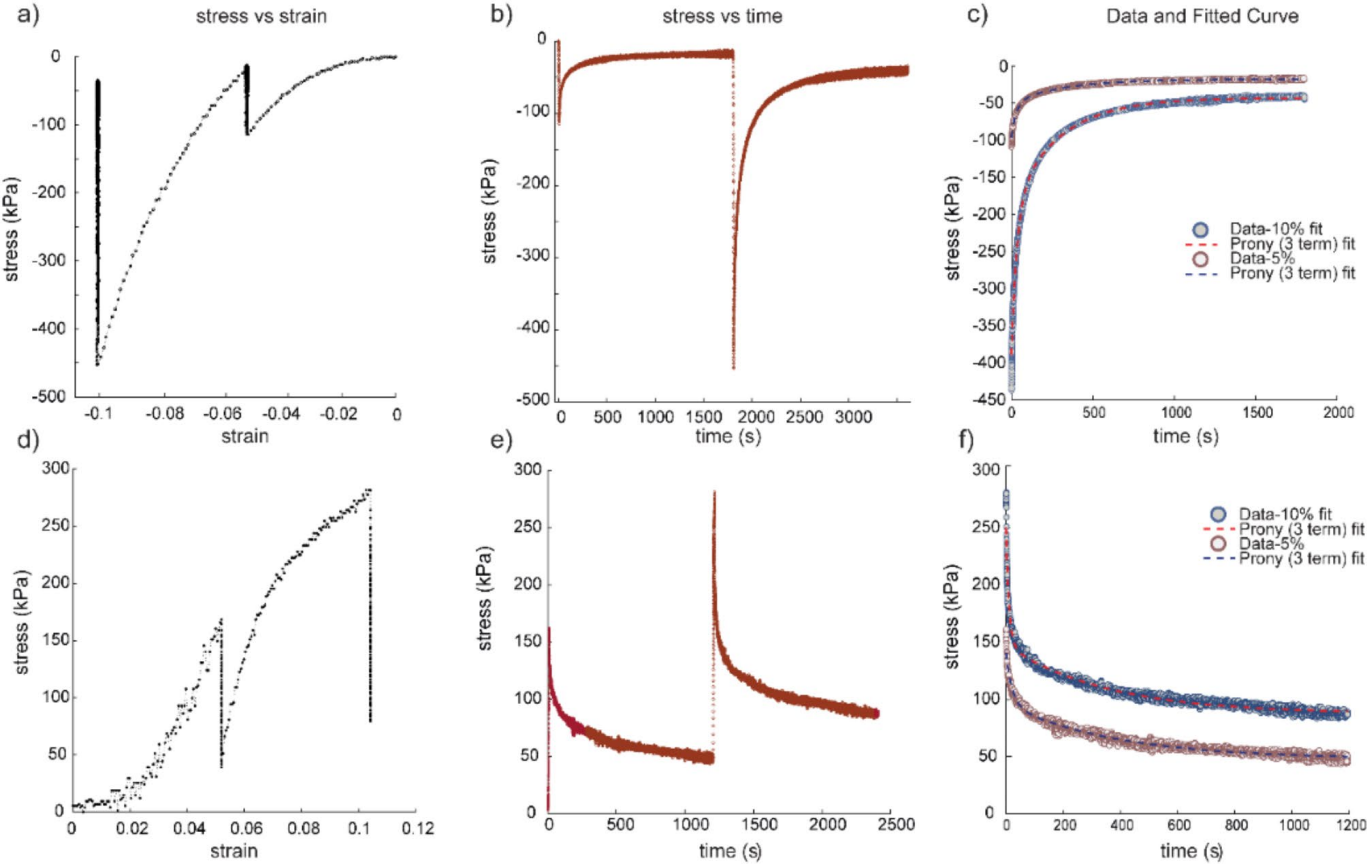
Representative stress-strain, stress-time profiles, and 3-parameter Prony series viscoelastic model fit to the experimental data under tensile and compressive loading. (a-c) A medial meniscus sample was tested under 5 and 10% compressive strain. (d-f) A lateral collateral ligament (LCL) sample was tested under 5 and 10% tensile strain.

**Fig. 6:**
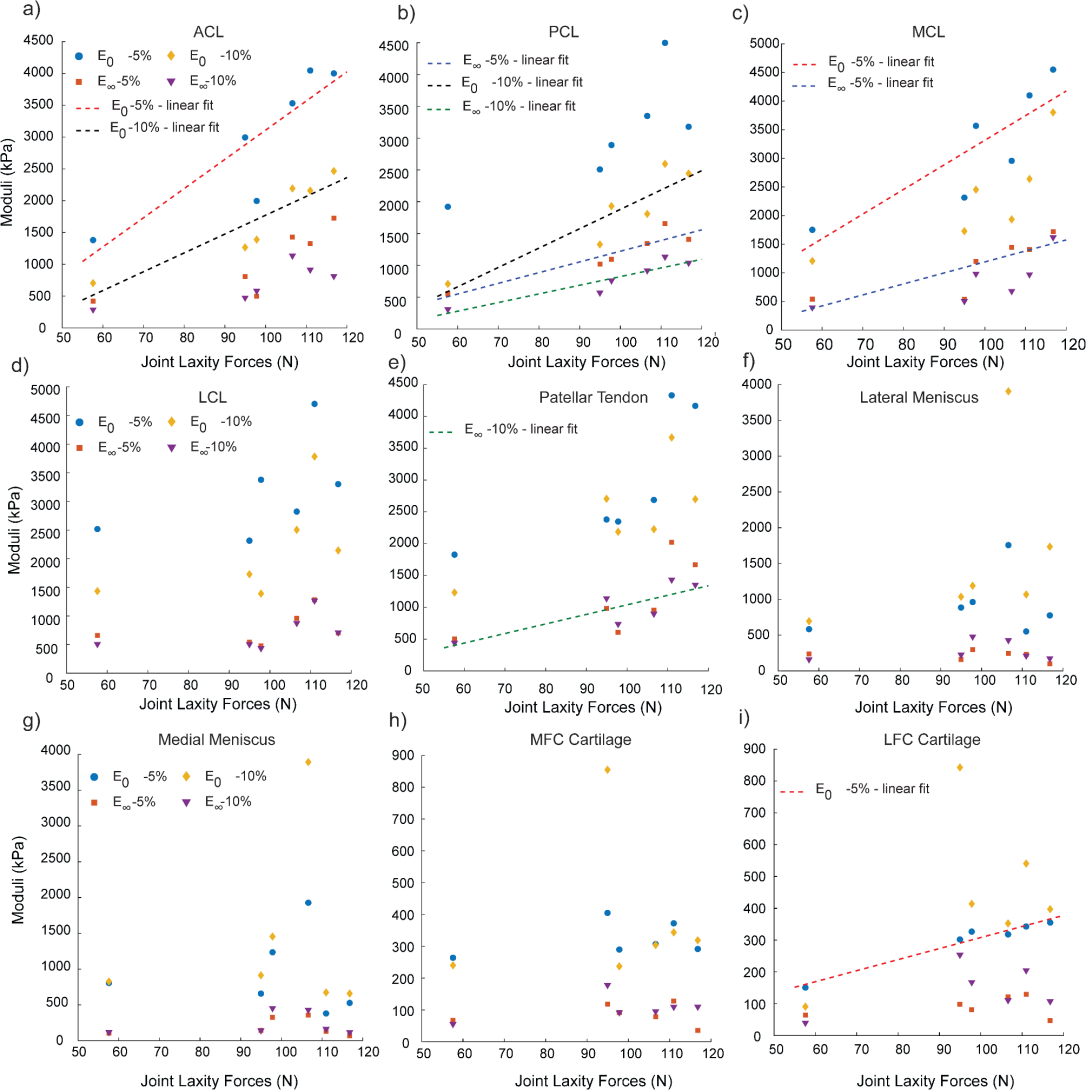
Variation in the tissue-specific viscoelastic properties (initial (E_0_) and relaxation (E_∞_) moduli (kPa) under 5 and 10% strain) for all the sets of tissues with the measured joint laxity forces (N). (a-e) Ligaments and tendons show a positive trend with the laxity forces whereas (f-i) menisci and cartilage moduli do not correlate with the joint laxity forces. The linear fits denote strong positive correlations (R^2^ > 0.7).

**Fig. 7:**
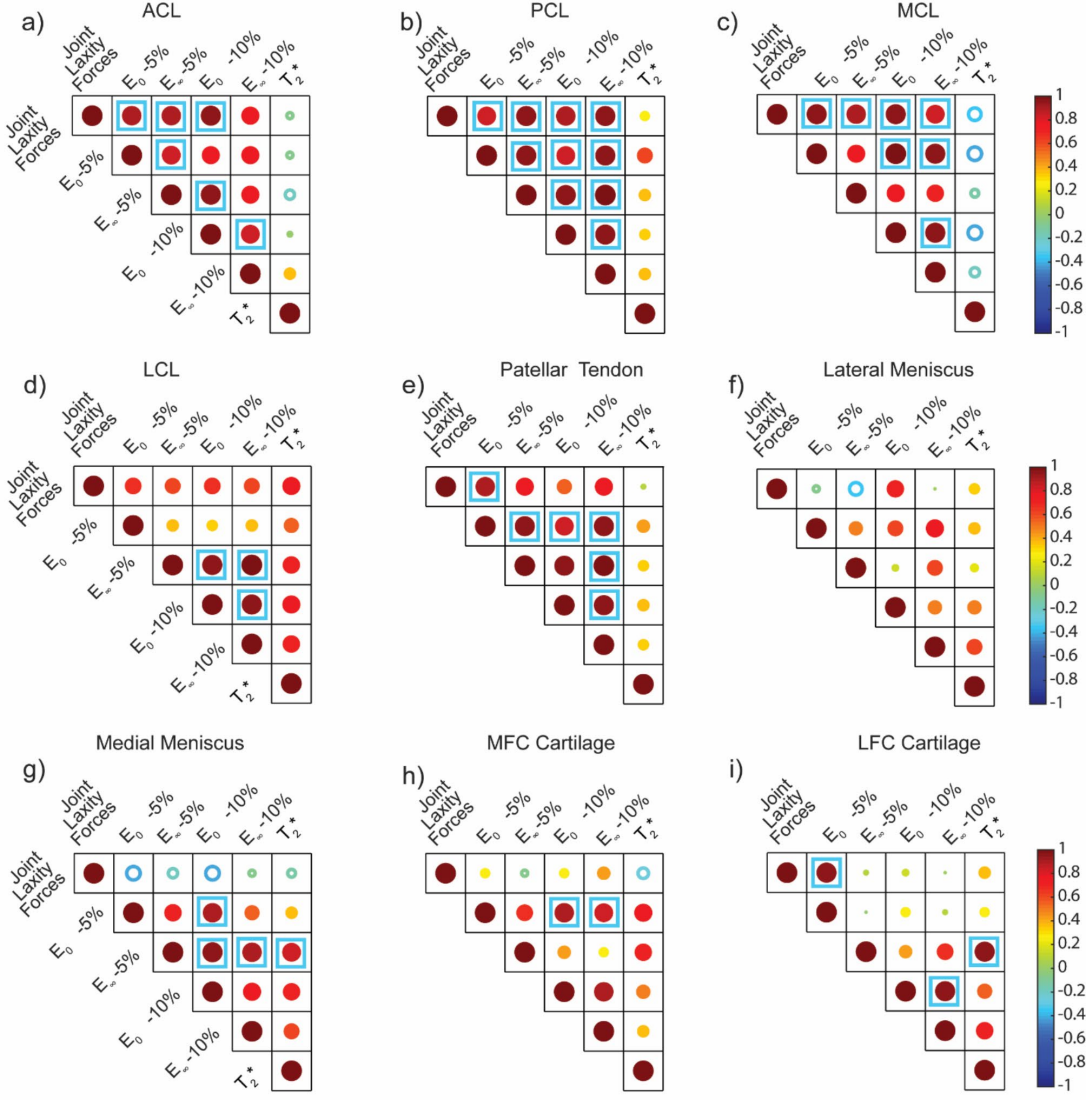
Correlation matrices plot for comparison among joint laxity forces (N), viscoelastic properties, and *T*_2_^*^ values for the (a-d) ligaments, (e) patellar tendon, (f-g) menisci, and (h-i) cartilage. Viscoelastic properties – initial (**E**_**0**_) and relaxation moduli (**E**_**∞**_)(kPa) – were evaluated under 5% and 10% strain. *T*_2_^*^ relaxation times (ms) were measured for individual tissues using MRI. The size and colour of the circles represent the Spearman’s rank correlation coefficient (ρ). Solid circles show a positive correlation (0 < ρ ≤ 1) and hollow circles show negative correlation (-1 ≤ ρ < 0). Strongly positive correlations (ρ ≥ 0.8) are denoted with a blue square. A corresponding table of correlation coefficients and p-values are provided in Supplemental Table 3.

We converted the load vs. time data from the stress relaxation experiments on the patellar tendon and the four ligaments (ACL, PCL, MCL, LCL) under tensile loading to stress vs. time data and used those to quantify initial modulus ($\:{E}_{o}$) and relaxation modulus ($\:{E}_{\infty\:}$) as measured under 5 and 10% strain (Fig. 5d-e). No grip failure or slippage was observed for any of the reported specimens during the tensile tests. The stress vs. time data was then fit to the 3-parameter Prony series model to obtain the tissue moduli and relaxation times (Fig. 5f). We found strong positive correlations using regression analysis between joint laxity forces and both $\:{E}_{o}$ and $\:{E}_{\infty\:}$ of the tendons and ligaments (R^2^ > 0.7) (Fig. 6a-e). Strong positive correlations (ρ>0.8) were obtained between the tissue moduli and joint laxity forces for all the ligaments and the patellar tendon (Fig. 7a-e), with statistically significant correlation coefficients for the MCL, PCL and ACL (Supplemental Table 3). We also found weak to moderate correlations with *T*_2_^*^, specifically for the LCL, PCL, MCL and the patellar tendon; however, they were not statistically significant (Fig. 7a-e, Supplemental Table 3). Relaxation time constants ($\:{\tau\:}_{min}$ and $\:{\tau\:}_{max}$) evaluated for all tested ligaments and tendon (Supplemental Table 4) but did not show statistically significant correlations to any other parameters.

### Joint laxity measurements do not show a significant correlation with viscoelastic properties of cartilage and meniscus

Following a similar procedure as with the tendons and ligament data, we converted the load vs. time data from the stress relaxation experiments on the femoral articular cartilage and menisci under compressive loading and used them to quantify the tissue $\:{E}_{o}$ and $\:{E}_{\infty\:}$ under 5 & 10% strain using a 3-parameter Prony series model (Fig. 5a-c). The corresponding relaxation time constants ($\:{\tau\:}_{min}$ and $\:{\tau\:}_{max}$) for the cartilage and menisci samples were determined (Supplemental Table 4). Unlike the tendons and ligaments, we did not observe any significant correlation with the whole-joint measurements and the cartilage and meniscus viscoelastic properties (Fig. 6f-i), except for the $\:{E}_{o}$ at 5% strain of articular cartilage samples extracted from the lateral femoral condyle (ρ = 0.04, *p* = 0.017). Articular cartilage extracted from both the lateral and medial femoral condyles showed strong positive correlation (ρ ≥ 0.8) between the initial and relaxation moduli and *T*_2_^*^ values (Fig. 7h-i). Specifically, statistically significant correlations were observed between $\:{E}_{0}$ at 10% and *T*_2_^*^ (ρ = 0.94, *p* = 0.017) for the lateral femoral condyle cartilage and for both $\:{E}_{0}$ and $\:{E}_{\infty\:}$ at 5% strain and *T*_2_^*^ (both ρ = 0.89, *p* = 0.033) for the medial femoral condyle cartilage. We also observed some strong and moderately positive correlations between the tissue moduli measured at 10% strain and the *T*_2_^*^ values for the lateral and medial menisci, although these correlations did not reach statistical significance (Fig. 7f-g, Supplemental Table 3).

## Discussion

The mechanical behavior of the knee depends on multiple parameters, ranging from the structure of the underlying bones, the structure and mechanics of soft tissues that support the joint, applied load, and motion constraints depending on the positioning of the joint^7^. Accordingly, using a combination of multiscale biomechanical testing and MRI, we have evaluated the correlations between whole joint and tissue level mechanics with joint geometry and tissue *T*_2_^*^ values in ovine specimens that provide useful insights into the knee joint mechanics facilitating data interpretation for clinical evaluation. We chose sheep due to multiple physiological similarities with humans including comparable body mass, similar anatomical structures, and equivalent distribution of mechanical loads acting across the joints^6,36^. Furthermore, their relative low-cost in comparison to other large animals and comparable bone healing rate to that of humans makes them a useful large animal model in the field of musculoskeletal diseases^6,37^.

We conducted joint-laxity studies on ovine stifles, measuring the range of forces resulting under cyclic displacements, and used anatomical MRI to quantify the epicondylar distance as a proxy for joint size. Joint laxity, which inversely relates to joint stiffness, has previously been linked with the stiffness of individual internal structures using theoretical studies^38^. However, these studies often employ computational approaches like lumped parameter models^31^ or finite element models^39^, which require detailed inputs on the structural and mechanical properties of the underlying structures, often based on experimental data derived from other studies. We sought to experimentally measure, in ovine stifles and individual tissue explants from the same specimen, the structural and mechanical properties necessary to form these connections at multiple length scales. To link joint size and laxity, we found a positive correlation between the epicondylar distance of the ovine stifles and the measured joint laxity forces. In a previous study^40^, statistical shape modelling showed that the morphological variabilities in the bone shape of tibia and femur and their relative alignment were linked with joint instability in human models. Our results therefore highlight the underlying correlations between joint anatomy and biomechanics, including the interdependencies between the knee joint size and its response to the external mechanical loading.

Furthermore, we also quantified the mechanical properties of individual tissues and investigated their correlations to the whole joint laxity response. The magnitude of loading rates as well as the choice of sample geometry can often influence the measurement of viscoelastic properties in soft tissues. As a result, the 0.05 mm/s loading rate chosen in our study due to experimental constraints, can be a factor impacting the obtained initial moduli for the tissues. While a faster loading rate may provide different values of initial moduli due to higher viscous effects, earlier reports have shown negligible influence of loading rates on the mechanical properties of tendon fascicles^41^, and tendon stiffness in humans measured in vivo^42,43^.

The experimentally obtained mechanical properties of the tissues obtained in our study are in good agreement with earlier published results. We observed that the range of the calculated moduli for the ovine patellar tendon and ligaments fell within 0.5–4.5 MPa in our study, which is similar to an earlier study on the elastic properties of bovine knee ligaments and patellar tendon^44^. The cartilage moduli were measured at up to 400 kPa, which is in line with published values reported for aggregate modulus of ovine fetal cartilage under compression extracted from the femoral condyles^45^. A potential future direction could include a regional map of mechanical properties throughout tissues of the joint, which could better support MRI-based computational models^27^. For the menisci, the initial modulus under compression is within the range 0.5-4 MPa, in agreement with compression moduli in ovine reported by Fischenich, et al.^46^. We found significantly positive correlations with the ligaments and patellar tendon moduli with the joint laxity forces. These results are in good agreement with previous studies^47,48^ that highlight that the knee joint responses are influenced by ligament mechanics. Further, in our study we observed the MCL to have the relatively highest initial modulus among the different ligaments and tendons, that aligns with an earlier study^28^ performed in bovine samples. While we do not see a significant correlation between the joint laxity forces and the cartilage and meniscus mechanics, these observations may depend on the type of loading applied. Ligaments are crucial for knee joint stability as they provide mechanical reinforcements and help in motion control^49^.

To provide noninvasive correlates to tissue content and ultrastructure, we measured *T*_2_^*^ of the different nonmineralized tissues of the stifle, including the ligaments, patellar tendon, menisci, and articular cartilage from the femoral condyles. The mean value of the *T*_2_^*^ relaxation times measured for different tissue types and are in line with previously published studies, although measurements from a 7T system will differ from those at different field strengths, such as 3T^50,51^. The high field MRI system enabled us to perform the *T*_2_^*^ quantification more accurately with reduced partial volume effects, which was important in identifying any correlations that may not be observable with lower resolution. Rough comparison with previously reported *T*_*2*_ values for similar large animals and human tissues also remains feasible, noting that, holding all else the same, *T*_2_^*^ is always faster than *T*_*2*_. A previous report showed a comparison of *T*_*1*_, *T*_*2*_, and *T*_2_^*^ mapping in articular cartilage in the shoulder resulting in similar trends and sensitivity to degeneration^52^. As *T*_2_^*^ mapping is faster than *T*_*2*_ mapping, for tissues with lower *T*_*2*_ values and faster signal decay, echo times may not be fast enough at our desired resolution for a fast-spin echo acquisition to achieve a good *T*_*2*_ fit (e.g., tendon, ligament) thereby making *T*_2_^*^ mapping a more viable option. *T*_2_^*^ mapping can also be incorporated into existing imaging protocols more effectively than T_1ρ_, which is not commercially available on many systems. While T_1ρ_ has been shown to be effective in cartilage, it is limited in its application to other joint tissues because it should be paired with an ultrashort echo time acquisition. Such custom sequences also have limited accessibility^53^ and are very specific to hardware configuration, restricting their usage, which was another reason to select *T*_2_^*^ analysis for our study. Nonetheless, analyzing additional quantitative MRI parameters would be useful in further understanding of the correlations with the individual tissue mechanics and may be a future direction of study.

We measured average *T*_2_^*^ values of the patellar tendon and ligaments of 10.68 ± 4.48 ms (tendon), 10.71 ± 4.19 ms (ACL), 8.15 ± 3.55 ms (MCL), 12.03 ± 5.16 ms (PCL), 9.51 ± 4.92 ms (LCL). These *T*_2_^*^ ranges are in general agreement with earlier studies performed at 3T in patellar tendon and cruciate ligaments from human cadavers^54^, Achilles tendon^23^ and ACLs^55^ in human subjects, and porcine ACLs^21,56^. Similarly, we found *T*_2_^*^ values for the lateral and media menisci were 8.07 ± 2.93 ms and 6.65 ± 2.55 ms, respectively, and in a similar range to previously reported values in sheep at 3T^57^ and goats at 9.4T^58^. We measured average *T*_2_^*^ values in articular cartilage from the lateral and medial femoral condyles of 14.95 ± 7.17 ms and 15.01 ± 6.79 ms. These values are comparable to prior *T*_2_^*^ studies at 7T^50,51^ in articular cartilage. Notably, these studies also demonstrate that *T*_2_^*^ is consistently lower at 7T than 3T in the same subjects, explaining the higher *T*_2_^*^ values (10–50 ms range) observed in articular cartilage from ovine^57^ and human^24^ sources.

Stress relaxation experiments were used to determine whether the viscoelastic properties, which are linked to tissue content, correlate to *T*_2_^*^ measurements. While we did not observe any significant correlations between the tissue moduli and the *T*_2_^*^ for the tendon and ligaments, there were some moderately positive correlations was observed between *T*_2_^*^ values and tissue moduli of the LCL and PCL, weak positive correlations for the patellar tendon and a weak inverse correlation for the MCL. These results are along the lines of previous studies that have found significant correlations between the *T*_2_^*^ relaxation time and tissue modulus in human patients with chronic patellar tendinopathy^59^. Another recent study in human athletes, has shown that while no correlations were observed between the Achilles tendon stiffness and young’s modulus with the *T*_2_^*^ long component, the *T*_2_^*^ short component was negatively correlated with tissue modulus^23^. On the other hand, we observed that the articular cartilage extracted from the medial and femoral condyles of the ovine stifles exhibited a strong (ρ ≥ 0.8) and significantly positive correlation between the tissue mechanics and the *T*_2_^*^ relaxation values. Although not statistically significant, strong and moderately positive correlations were observed between the mechanical properties of menisci and the corresponding *T*_2_^*^ as well. Mechanical properties of soft tissues like articular cartilage and meniscus have been shown to correlate with *T*_*2*_ relaxation time in human patients^60^. Additionally, the viscoelastic properties of cartilage have been previously correlated with tissue degradation and early OA progression using canine cranial cruciate ligament transection models^61^; however, the correlations between the tissue viscoelastic properties with relaxometry methods such as *T*_2_^*^ remain an area for future work^62^. Although we did not see any significant correlation between the tissue specific *T*_2_^*^ values with the joint laxity forces (Fig. 7), some weak and moderately positive correlations were observed for LCL (ρ = 0.77, *p* = 0.103), cartilage from the lateral femoral condyles (ρ = 0.37, *p* = 0.49), and lateral menisci (ρ = 0.31, *p* = 0.56); and a weak negative correlation for MCL (ρ=-0.37, *p* = 0.49).

While increasing the sample size of our study could further improve the robustness of the measured statistical correlations between the tested metrics, *Post hoc* statistical analysis demonstrated that the number of specimens tested in this study was a sufficient sample size^63,64^ for obtaining strongly positive correlation strengths (ρ ≥ 0.89). Our results also corroborate with studies that suggest correlations between joint laxity and the *T*_2_^*^ relaxation time for specific tissues like cartilage and meniscus in context to joint health^65,66^. Correlations between tissue mechanics and *T*_2_^*^ are affected by variations to both types of parameters. In addition to the differences in magnetic field strengths and field inhomogeneity, variation in tissue magnetic properties is also dependent on various factors such as the sample size, the animal age, sex, body weight, scanning conditions, and other parameters^51,67^. Along these lines, we observe a moderate and weak negative correlation between animal age and both joint laxity forces (ρ=-0.74, *p* = 0.12) and epicondylar distance (ρ=-0.5, *p* = 0.33), respectively, for the specimens in our study (Supplemental Fig. 2). All MRI measurements were performed within the same freeze-thaw cycle as laxity experiments before which they were stored in -20ºC. Previous studies have shown that mean *T*_*2*_ or *T*_2_^*^ values after unaffected by low numbers of freeze-thaw cycles. Human Achilles tendon specimens are not significantly altered until 5 freeze-thaw cycles^68^, and no notable changes in porcine articular cartilage *T*_*2*_ were detectable within a single freeze–thaw cycle^69^. Therefore, we expected a negligible impact of the storage conditions on the measured *T*_2_^*^ values. Variations in *T*_2_^*^ values could also arise because of differences in positioning of the knee during scanning^70^. To reduce such variabilities in the measurements, we maintained a similar positioning of the ovine stifle joints for all the specimens during scanning. However, because the scans were performed in the intact joint, the differences in orientation of the primary direction of collagen fibre alignment in the tissues, with respect to the direction of the main magnetic field, cannot be fully controlled.

In conclusion, using a multiscale approach, we found correlations between whole joint mechanics, joint size, and individual tissue properties in ovine stifles in this study, which may improve our understanding of the knee physiology. At the whole joint level, we performed joint laxity experiments to measure the biomechanical properties, and used anatomical MRI scans to measure epicondylar distance as a metric for joint morphology At the tissue level, we conducted stress relaxation tests to measure the viscoelastic properties of the ligaments (ACL, PCL, MCL, LCL), the patellar tendon, menisci and femoral cartilage and also used quantitative MRI scans to measure the tissue specific *T*_2_^*^ values. We calculated the correlation coefficients between these metrics measured at whole joint and tissue level. Specifically, we show that the forces measured during whole joint testing correlated directly with femur size, measured using the epicondylar distance. We also find that the viscoelastic properties of the tendons and ligaments correlated positively with joint laxity forces. We also observe a weak to moderate positive correlation with tissue viscoelastic properties with *T*_2_^*^ for patellar tendon, PCL and LCL, weak negative correlation with MCL and significant positive correlations for cartilage samples from the femoral condyles. However, we did not find any other significant correlations between tissue *T*_2_^*^ relaxation times and viscoelastic properties for the other groups of tissues. Our results on the correlations between soft tissue biomechanics measured ex-vivo and *T*_2_^*^ can be used as preliminary data that can later be further extended to studying the biomechanics and physiological properties of knee joints. These results also provide useful insights into the differential role of individual tissue properties that can be further analyzed using a Bayesian modelling framework to explore the relative effect and contribution of individual metrics to the overall joint behavior; and can be used to develop subject-specific computational models to assess the knee joint responses. This will facilitate modelling the knee joint as an ensemble of the underlying soft structures, where all the inputs including tissue material properties, joint morphology, loading conditions are derived from a same cohort using consistent test conditions. Such models will have improved accuracy in predicting the joint responses in both healthy aging or under specific disease, contributing to development of better diagnostic techniques and clinical assessment.

## Methods

### Ovine stifle collection and storage

Ovine stifles were obtained after humane euthanasia of sheep (barbiturate overdose via intravenous injection) used for unrelated teaching purposes before preparation for multi-scale experiments (Fig. 1). Collection of tissues under these animal protocols were performed according to the relevant guidelines and regulations. All experiments described herein were performed postmortem on tissues collected. A total of six (two male (S-2 and S-5) and four female (S-1, S-3, S-4 and S-6)) sheep were included in our study. All animals were over 24 months old, falling within an age range of 2–5 years. Ages of each sheep were estimated from their teeth and are approximated as 25 (2 sheep), 37 (2 sheep), 49 and 61 months (1 each). Skin and most of the musculature were removed except for those connected to the quadriceps and patellar tendon, to keep the joint capsule intact and not compromise with the joint stability. The distal femur and proximal tibia were cut mid-diaphysis and cleaned for ease of potting during whole joint experiments. The collected stifles were cleaned, wrapped in cotton gauze soaked in phosphate-buffered saline (PBS) to maintain hydration, and stored in plastic bags at -20℃. The frozen ovine stifles were thawed at 4℃ for 24–36 h before mechanical testing and MRI.

### Joint laxity testing

Joint laxity testing (Fig. 1) was performed on ovine stifles using a 10-kN capacity load frame (MTS Systems). Prior to mechanical testing, the distal end of the femur and the proximal end of the tibia were cleared of tissue and embedded in cylindrical aluminum tubes using polymethyl methacrylate (Coralite Dental Manufacturing), mixed at 3:1 powder-to-liquid ratio and cured for 30 min. The stifle was wrapped in PBS-soaked gauze to prevent dehydration of the tissues during the curing time. The potted joints were attached to custom-built testing fixtures, coupled with a pair of orthogonal screws to prevent off-axis motions (Fig. 2a) and mounted on the load frame at a 90° flexion angle, verified using a 360 ° Goniometer^71^. The use of orthogonal screws, to prevent off-axis motions and slippage during testing, can induce off-axis forces, however the magnitude of such forces was much smaller in comparison to the joint laxity forces and no visible deformation of the joint was observed at the end of the testing. The load frame was adjusted to ensure that both the testing fixtures were at the sample plane and horizontally offset to prevent any off-axis motions during the tests. After mounting the joint on the load frame, at the desired 90° flexion angle, without applying any loads, the displacement and force readings were reset, and this configuration was considered as the neutral position of the joint. Each stifle was preconditioned for twenty cycles using displacement control mode, and triangular displacements of ± 0.5 mm was provided at a rate of 0.2 mm/s^9^. Following preconditioning, the joint was allowed to rest in its neutral position for two minutes and then loaded cyclically to ± 1.5-mm triangular displacements for 10 cycles at 0.2 mm/s (Fig. 2b). The difference between the peak forces at the endpoints of the cyclic displacement input was calculated as the range of forces experienced during testing and was used as a measure of joint laxity.

### MRI for anatomy and *T*_2_^*^ mapping

After the joint laxity tests, the ovine stifles were imaged (Fig. 1) using a Bruker Biospec 70/30 7T MRI (Billerica, MA) running manufacturer’s software (ParaVision 6.0.1). To properly fit the specimens within the 7T system, excess bone and potting material were removed for MRI and the samples were kept in an extended position during imaging. Both MRI scans and the joint laxity tests were performed within the same freeze thaw cycle. Each stifle was wrapped in gauze soaked in 1x PBS gauze to keep a hydrated environment during scanning. 3D *T*_*1*_-weighted FLASH scans [8-ms echo time (TE), 50-ms repetition time (TR), 20° flip angle, voxel size = 1/3 mm isotropic] were acquired to visualize tissue geometry (Fig. 2d). A 2D multi-slice multi-echo gradient echo sequence was used to determine the *T*_2_^*^ relaxation value of the tissues [TEs at 3.5 to 58.5 ms, at 5 ms echo spacing, TR = 1500 ms, in-plane spatial resolution = 1/3 mm × 1/3 mm, flip angle = 50^o^, slice thickness = 1 mm]. Joint anatomy was manually segmented in 3D Slicer (Version 5.2.2) software^72^, and the femoral epicondyle-to-epicondyle distance was determined using this software (Fig. 2e). Inter-epicondylar distance has previously been established as a reliable 2-dimensional anatomical marker in multiple clinical applications, such as the determination of joint line location during revision knee arthroplasty^73^ the measurement of graft length for anterior cruciate ligament reconstruction (ACLR) techniques^74^, estimation of total femoral cartilage area etc., and hence was used as a metric for overall joint morphology in our study. The *T*_2_^*^ analysis was performed using all the acquired slices from the 3-D scans of individual tissues for each medial meniscus, lateral meniscus, articular cartilage from the medial and lateral femoral condyles, the anterior and posterior cruciate ligaments (ACL, PCL), medial and lateral collateral ligaments (MCL, LCL), and the patellar tendon. The signal intensity from the multi-TE images were fit to equation $\:S={S}_{0}{e}^{-\frac{TE}{{T}_{2}^{*}}}$ to estimate *T*_2_^*^ relaxation times in MATLAB (Version 2022B. Natick, MA). $\:{S}_{0}$ represents the initial signal intensity at time t = 0, and the equation describes the exponential decay of the signal intensity with time as a function of the *T*_2_^*^. Reported *T*_2_^*^ averages include all pixels that had an exponential fit with an R^2^ value above 0.7 (Supplemental Fig. 1).

### Tissue-specific mechanical testing to quantify material properties

Following the whole joint testing and MRI, each stifle was dissected to extract individual tissues for mechanical testing (Fig. 1). Tissue specimens of standardized geometries suitable for mechanical testing were dissected from the ligaments (ACL, PCL, MCL, LCL) the patellar tendon, menisci, and articular cartilage from the femoral condyles of the tested ovine stifles. Prior to mechanical testing, tissue explants were equilibrated in PBS supplemented with 10 µL/mL protease inhibitor cocktail (Halt™, Thermo Fisher Scientific) and 10 µL/mL 0.5-M EDTA (Thermo Fisher Scientific) at 4℃ overnight.

For the cartilage and meniscus, cylindrical shaped explants were obtained using a bone punch (6-mm diameter) from the central location of the femoral condyles, perpendicular to the tibial surface and a razor blade was used to trim to even thickness (~ 1 mm thickness for articular cartilage, and ~ 3 mm thickness for meniscus) before compression testing^75,76^. For the cartilage specimens, the articular surface and the full superficial zone was kept intact, only a small portion of the deep zone and the full calcified zone and the subchondral bone layers removed. The thickness of the tissue specimens was measured using a digital caliper. Additionally, the trimmed specimens were placed between two parallel glass slides using a custom set-up and were visually inspected to confirm uniform thickness. Cartilage and menisci specimens were adhered to a 35-mm petri dish using a single drop of cyanoacrylate (~ 10 µL volume) and submerged in a PBS bath during testing. A preload of 0.1 N was applied on both the cartilage and meniscus samples to ensure proper contact between the tissue specimen and the compression platen. Subsequently, the force and displacement values were then zeroed, and the experimental measurements were recorded. After preconditioning of 20 cycles to 5% compressive strain, at a rate of 1%/s of the gauge length^77,78^, stress relaxation experiments were performed on the tissue specimens at 5 and 10% compressive strain using a cylindrical compression platen (10 mm diameter) to quantify viscoelastic behavior^79^. The dwell time was kept at 30 min at each strain step for the explants to reach a steady state^80^.

Tendon and ligament specimens were dissected to a target gauge length of ~ 10 mm, width of ~ 5 mm, and thickness of ~ 2 mm, from the central region of the tissues^28^, such that the collagen fibers were aligned along the longitudinal direction of the samples. Sample cross sections were measured using a digital caliper at multiple points along the length of the specimen to ensure uniform width and thickness. Tensile tests were performed on a TA Electroforce 3230 with custom grips suitable for tissue testing in both submersible and non-submersible conditions. The grip faces were knurled to prevent slippage during testing and were also covered using sandpaper, adhered to the grips to increase friction between the sample and the claps and prevent slippage. Preconditioning of 20 cycles to 5% tensile strain at 0.05 mm/s^81,82^ was conducted. All strain measurements were performed based on the grip-to-grip actuator displacement and tissue gauge length. Stress relaxation tests were then performed to 5 and 10% tensile strain, with a dwell time of 20 min^83^, to quantify their viscoelastic properties^84^.

### Estimation of tissue viscoelastic parameters from experimental data

The load vs. time data obtained from the stress relaxation tests was converted to the corresponding stress vs. time data using the information about sample geometry for each specimen. These data sets were used to estimate the viscoelastic properties of the explants using a 3-parameter nonlinear Prony Series model^84^:

$$\:\begin{array}{c}\sigma\:\left(t\right)={\sigma\:}_{\infty\:}+{\sigma\:}_{1}{e}^{-\left(\frac{t}{{\tau\:}_{1}}\right)}+{\sigma\:}_{2}{e}^{-\left(\frac{t}{{\tau\:}_{2}}\right)}\: \quad \left(1\right)\end{array}$$

$\:{\sigma\:}_{\infty\:}$, $\:{\sigma\:}_{1}$, and $\:{\sigma\:}_{2}$ are stress parameters and $\:{\tau\:}_{1}$ and $\:{\tau\:}_{2}$ are their respective relaxation time constants. Parameters from the Prony series model were used to quantify the initial modulus ($\:{E}_{0}$)

$$\:\begin{array}{c}{E}_{0}=\frac{{\sigma\:}_{\infty\:}+{\sigma\:}_{1}+{\sigma\:}_{2}}{\epsilon\:}\: \quad \left(2\right)\end{array}$$

and relaxation modulus ($\:{E}_{\infty\:}$)

$$\:\begin{array}{c}{E}_{\infty\:}=\frac{{\sigma\:}_{\infty\:}}{\epsilon\:}\: \quad \left(3\right)\end{array}$$

where $\:\epsilon\:$ is the applied strain^85^. In our analysis, we have defined the shorter time constant among $\:{\tau\:}_{1}$ and $\:{\tau\:}_{2}$ as $\:{\tau\:}_{min}$ and the longer one as $\:{\tau\:}_{max}$. The Prony series model was fitted to experimental data using a non-linear least squares method in MATLAB. All models fit to experimental data reported in this study had R^2^ > 0.7.

### Statistical analysis

All data are reported as mean ± standard deviation, unless otherwise indicated, and are publicly available for access^86^. Statistical analysis was performed using MATLAB, with a statistical significance defined at *p* < 0.05 for all hypothesis tests. The p-values were quantified using the *corrplot* function for two-tailed tests for the correlation coefficients. To quantify the strength of the correlations between different metrics, we used Spearman’s correlation, a non-parametric measure of rank correlation and quantified the Spearman’s rank correlation coefficient (ρ) and the corresponding p-values between the joint laxity forces and the epicondyle-to-epicondyle distance, between normalized joint laxity forces and tissue moduli, and the *T*_2_^*^ relaxation times. All R^2^ values reported in this study are obtained by regression analysis. In our study, we have regarded r values between 0.2 and 0.39 as weak, 0.40–0.59 as moderate, 0.6–0.79 as strong and 0.8-1 as significantly strong correlation^87^. *Post hoc* statistical analysis to determine necessary sample size for obtaining strongly positive correlation strengths (ρ ≥ 0.89, were calculated using Fisher Z transformation^88^ to obtain 5% significance level test (α = 0.05) and 80% power (β = 0.2).

## Electronic supplementary material

Below is the link to the electronic supplementary material.

Supplementary Material 1

Supplementary Material 2

Supplementary Material 3

Supplementary Material 4

Supplementary Material 5

Supplementary Material 6

## Data Availability

All data reported in this manuscript are are publicly available for access in the Harvard Dataverse repository via https://doi.org/10.7910/DVN/9MAQDP.
