# Supplementary material for: Multiscale correlations between joint and tissue-specific biomechanics and anatomy in postmortem ovine stifles
Source: Sci Rep. 2025 Feb 7;15:4630. doi: 10.1038/s41598-025-87491-w (PMC11806062; doi:10.1038/s41598-025-87491-w)
Supplement: Supplementary file 1 — Supplementary Material 1 [file 41598_2025_87491_MOESM1_ESM.docx]

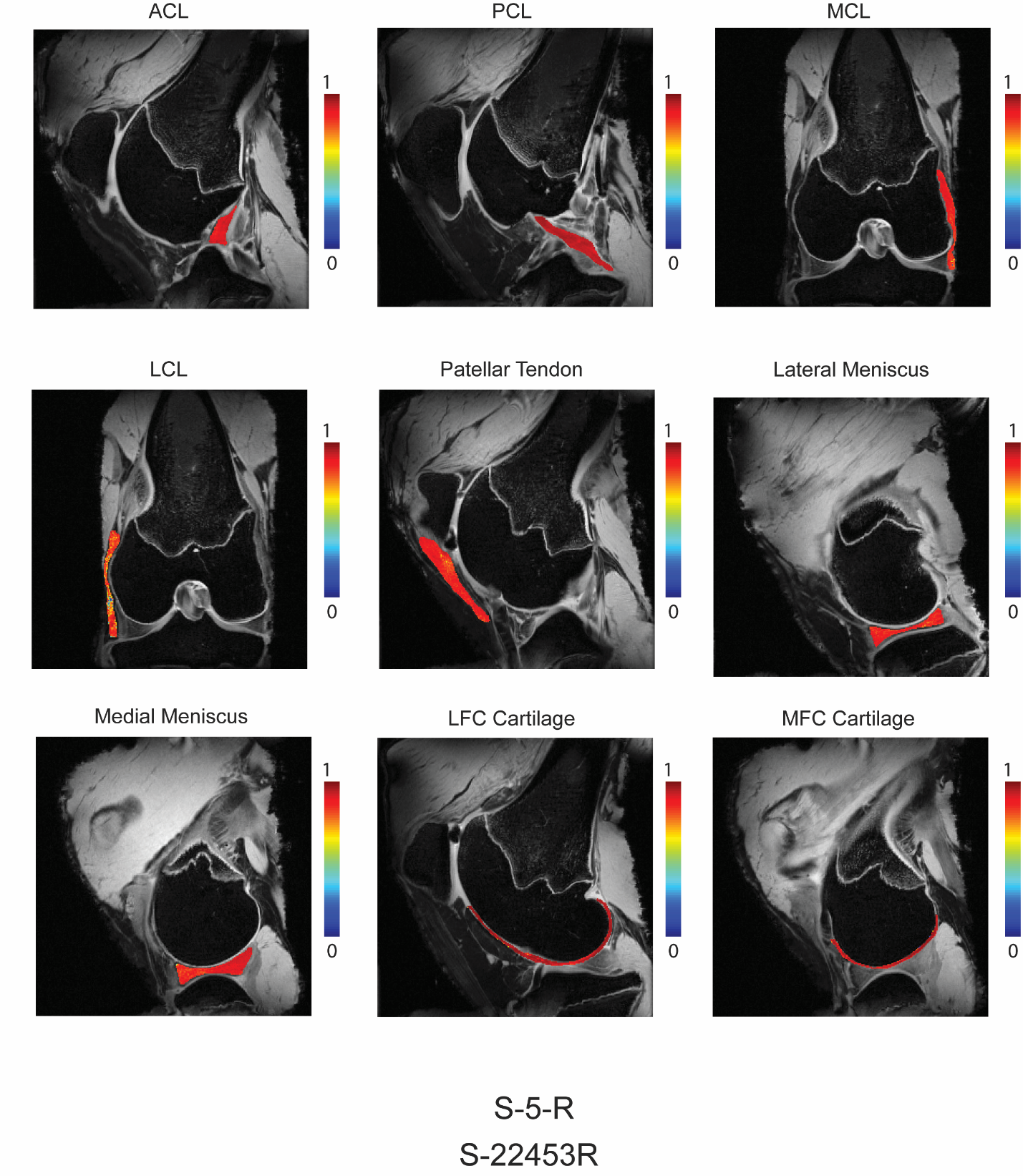


**Supplementary Figure 1.** ***R* ^2^ maps for the corresponding T*_2_* ^*^** **calculated from 2D multi-slice *T_2_ ^*^*** **multi gradient echo sequence and shown in representative slices for each tissue.** Slices that show the *R^2^* map for the anterior cruciate ligament (ACL), posterior cruciate ligament (PCL), medial collateral ligament (MCL), lateral collateral ligament (LCL), patellar tendon, lateral and medial menisci, and cartilage from the lateral femoral condyle (LFC) and media femoral condyle (MFC) are shown for a representative stifle.
