# Supplementary figures and images for: Multiscale correlations between joint and tissue-specific biomechanics and anatomy in postmortem ovine stifles
Source: Sci Rep. 2025 Feb 7;15:4630. doi: 10.1038/s41598-025-87491-w (PMC11806062; doi:10.1038/s41598-025-87491-w)

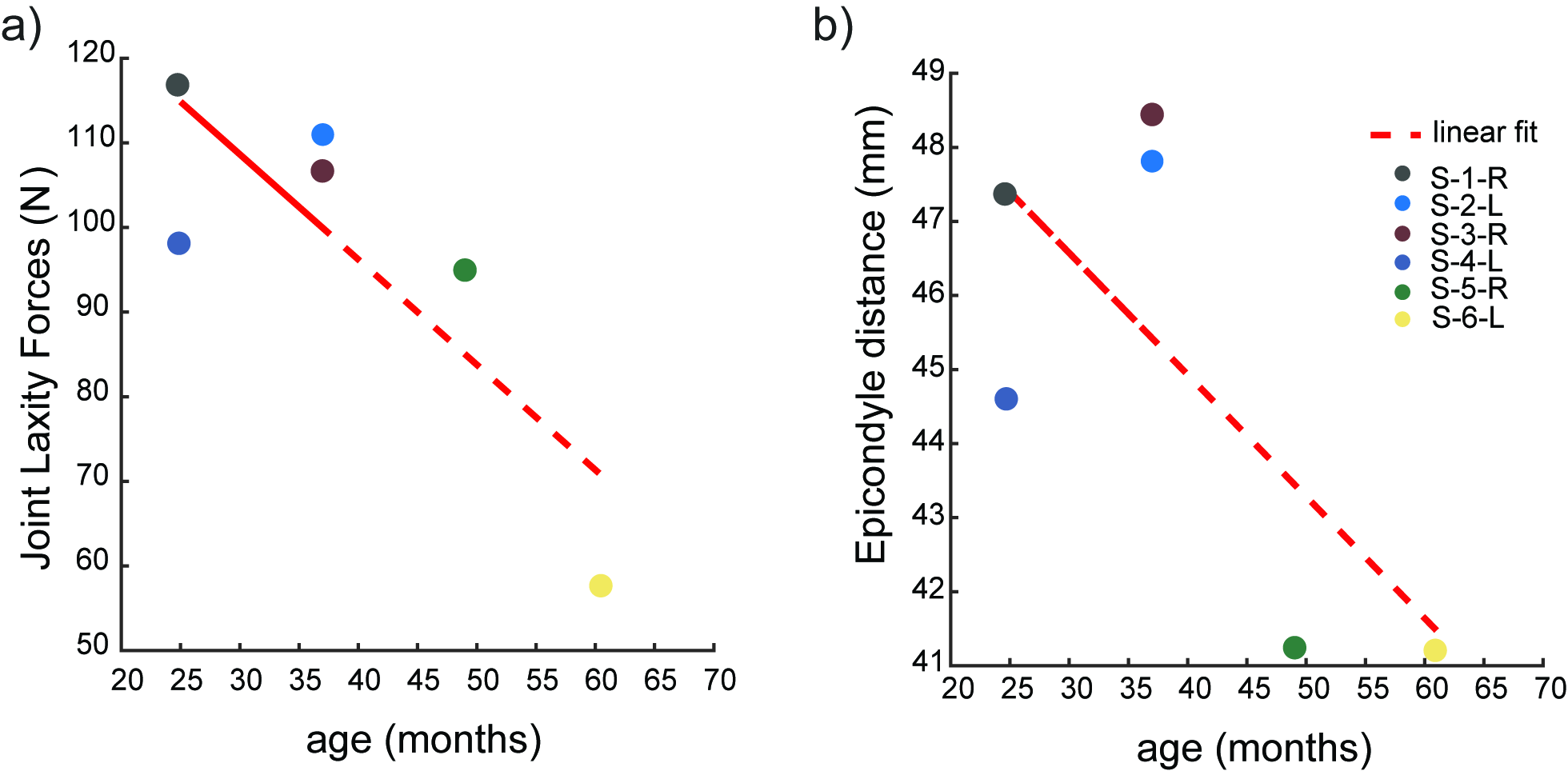

Supplement: Supplementary file 2 — Supplementary Material 2 [file 41598_2025_87491_MOESM2_ESM.tif]
