# Supplementary material for: Multiscale correlations between joint and tissue-specific biomechanics and anatomy in postmortem ovine stifles
Source: Sci Rep. 2025 Feb 7;15:4630. doi: 10.1038/s41598-025-87491-w (PMC11806062; doi:10.1038/s41598-025-87491-w)
Supplement: Supplementary file 3 — Supplementary Material 3 [file 41598_2025_87491_MOESM3_ESM.docx]

**Supplemental Table 1. Magnitude of joint forces in the anterior direction and in the posterior direction for all tested ovine specimens. The sex of each specimen is also provided, M- Male and F-Female.**

| **Specimen** | **Force (N)** | **Anterior Direction** | **Posterior direction** |
| --- | --- | --- | --- |
| ***S-1 (F)*** | 116.782 | 43.5405 | -73.2416 |
| ***S-2 (M)*** | 110.976 | 66.826 | -41.15 |
| ***S-3 (F)*** | 106.6 | 50.0995 | -56.5 |
| ***S-4 (F)*** | 97.8139 | 52.9749 | -44.839 |
| ***S-5 (M)*** | 94.961 | 50.7306 | -44.2306 |
| ***S-6 (F)*** | 57.588 | 23.3064 | -34.2816 |
