# Supplementary material for: Multiscale correlations between joint and tissue-specific biomechanics and anatomy in postmortem ovine stifles
Source: Sci Rep. 2025 Feb 7;15:4630. doi: 10.1038/s41598-025-87491-w (PMC11806062; doi:10.1038/s41598-025-87491-w)
Supplement: Supplementary file 4 — Supplementary Material 4 [file 41598_2025_87491_MOESM4_ESM.docx]

**Supplemental Table 2. *T_2_^*^* relaxation values in ms (mean ± std) for the menisci, cartilage (from either medial and lateral femoral condyles), ligaments and tendon for the tested ovine specimens (total six – one stifle per each animal). The sex of each specimen is also provided, M- Male and F-Female.**

| **Specimen** | **Lateral Meniscus** | **Medial Meniscus** | **Cartilage (LFC)** | **Cartilage (MFC)** | **ACL** | **MCL** | **PCL** | **LCL** | **Patellar Tendon** |
| --- | --- | --- | --- | --- | --- | --- | --- | --- | --- |
| **S-1 (F)** | 6.77 ±2.66 | 5.51±1.81 | 11.45±6.4 | 10.48±5.61 | 7.85±2.73 | 6.47±2.11 | 8.46±4.19 | 9.64±5.15 | 7.16±3.27 |
| **S-2 (M)** | 9.1±3.71 | 6.98±2.76 | 19.49±10.5 | 15.87±6.25 | 11.22±4.35 | 5.95±2.28 | 12.95±6.08 | 10.44±6.07 | 16.66±6.92 |
| **S-3 (F)** | 9.45±3.81 | 7.34±3.39 | 19.28±9.14 | 16.88±6.46 | 12.97±5.58 | 11.17±6.95 | 16.99±6.79 | 13.78±8.91 | 14.97±6.17 |
| **S-4 (F)** | 8.15±2.36 | 6.9±2.46 | 14.33±4.04 | 15.43±6.55 | 13.13±4.58 | 9.28±3.38 | 13.58±6.1 | 8.26±3.11 | 6.52±2.76 |
| **S-5 (M)** | 8.63±2.87 | 7.31±2.07 | 15.13±6.69 | 20.25±8.5 | 10.67±3.81 | 9.14±3.81 | 12.14±3.67 | 7.66±2.21 | 8.51±2.59 |
| **S-6 (F)** | 6.32±2.16 | 5.89±2.82 | 10.06±6.22 | 11.15±7.4 | 8.44±4.06 | 6.89±2.74 | 8.09±4.09 | 7.29±4.09 | 10.28±5.17 |
