# Supplementary material for: Multiscale correlations between joint and tissue-specific biomechanics and anatomy in postmortem ovine stifles
Source: Sci Rep. 2025 Feb 7;15:4630. doi: 10.1038/s41598-025-87491-w (PMC11806062; doi:10.1038/s41598-025-87491-w)
Supplement: Supplementary file 5 — Supplementary Material 5 [file 41598_2025_87491_MOESM5_ESM.docx]

**Supplemental Table 3. Tissue-specific** **Spearman’s rank correlation coefficient (****ρ) and p-values calculated from comparison between joint forces (N), viscoelastic properties (instantaneous (**$E_{o}$**) and relaxation (**$E_{\infty}$**) moduli (kPa) under 5 & 10% strain), and *T_2_^*^* relaxation times (ms).** The upper diagonal terms represent the correlation coefficient (**ρ**), and the lower diagonal terms represent the corresponding p-values (in italics). Significant p-values and their corresponding correlation coefficients are underlined.

| **Anterior Cruciate Ligament** | | | | | | |
| --- | --- | --- | --- | --- | --- | --- |
| **ρ**  **p** | **Joint Forces (N)** | $\boldsymbol{E}_{\boldsymbol{o}}$**, -5%** | $\boldsymbol{E}_{\boldsymbol{\infty}}$**, -5%** | $\boldsymbol{E}_{\boldsymbol{o}}$**, -10%** | $\boldsymbol{E}_{\boldsymbol{\infty}}$**, -10%** | **Average *T_2_^*^* (ms)** |
| **Joint Forces (N)** | 1 | 0.89 | 0.89 | 0.94 | 0.77 | -0.09 |
| $\boldsymbol{E}_{\boldsymbol{o}}$**, -5%** | *0.03* | 1 | 0.83 | 0.77 | 0.77 | -0.09 |
| $\boldsymbol{E}_{\boldsymbol{\infty}}$**, -5%** | *0.03* | *0.058* | 1 | 0.94 | 0.77 | -0.2 |
| $\boldsymbol{E}_{\boldsymbol{o}}$**, -10%** | *0.017* | *0.1* | *0.017* | 1 | 0.83 | -0.03 |
| $\boldsymbol{E}_{\boldsymbol{\infty}}$**, -10%** | *0.1* | *0.1* | *0.103* | *0.058* | 1 | 0.37 |
| **Average *T_2_^*^* (ms)** | *0.92* | *0.92* | *0.713* | *0.497* | *0.414* | 1 |
|  |  |  |  |  |  |  |
| **Posterior Cruciate Ligament** | | | | | | |
| **ρ**  **p** | **Joint Forces (N)** | $\boldsymbol{E}_{\boldsymbol{o}}$**, -5%** | $\boldsymbol{E}_{\boldsymbol{\infty}}$**, -5%** | $\boldsymbol{E}_{\boldsymbol{o}}$**, -10%** | $\boldsymbol{E}_{\boldsymbol{\infty}}$**, -10%** | **Average *T_2_^*^* (ms)** |
| **Joint Forces (N)** | 1 | 0.83 | 0.94 | 0.89 | 0.94 | 0.25 |
| $\boldsymbol{E}_{\boldsymbol{o}}$**, -5%** | *0.058* | 1 | 0.94 | 0.83 | 0.94 | 0.6 |
| $\boldsymbol{E}_{\boldsymbol{\infty}}$**, -5%** | *0.017* | *0.017* | 1 | 0.94 | 0.99 | 0.37 |
| $\boldsymbol{E}_{\boldsymbol{o}}$**, -10%** | *0.033* | *0.058* | *0.017* | 1 | 0.94 | 0.31 |
| $\boldsymbol{E}_{\boldsymbol{\infty}}$**, -10%** | *0.017* | *0.017* | *0.003* | *0.017* | 1 | 0.37 |
| **Average *T_2_^*^* (ms)** | *0.658* | *0.658* | *0.497* | *0.564* | *0.497* | 1 |
|  |  |  |  |  |  |  |
| **Lateral Collateral Ligament** | | | | | | |
| **ρ**  **p** | **Joint Forces (N)** | $\boldsymbol{E}_{\boldsymbol{o}}$**, -5%** | $\boldsymbol{E}_{\boldsymbol{\infty}}$**, -5%** | $\boldsymbol{E}_{\boldsymbol{o}}$**, -10%** | $\boldsymbol{E}_{\boldsymbol{\infty}}$**, -10%** | **Average *T_2_^*^* (ms)** |
| **Joint Forces (N)** | 1 | 0.66 | 0.6 | 0.66 | 0.6 | 0.77 |
| $\boldsymbol{E}_{\boldsymbol{o}}$**, -5%** | *0.175* | 1 | 0.37 | 0.31 | 0.37 | 0.54 |
| $\boldsymbol{E}_{\boldsymbol{\infty}}$**, -5%** | *0.241* | *0.497* | 1 | 0.94 | 0.99 | 0.71 |
| $\boldsymbol{E}_{\boldsymbol{o}}$**, -10%** | *0.175* | *0.564* | *0.017* | 1 | 0.94 | 0.77 |
| $\boldsymbol{E}_{\boldsymbol{\infty}}$**, -10%** | *0.242* | *0.497* | *0.002* | *0.017* | 1 | 0.71 |
| **Average *T_2_^*^* (ms)** | *0.103* | *0.297* | *0.136* | *0.103* | *0.1136* | 1 |
|  |  |  |  |  |  |  |
| **Medial Collateral Ligament** | | | | | | |
| **ρ**  **p** | **Joint Forces (N)** | $\boldsymbol{E}_{\boldsymbol{o}}$**, -5%** | $\boldsymbol{E}_{\boldsymbol{\infty}}$**, -5%** | $\boldsymbol{E}_{\boldsymbol{o}}$**, -10%** | $\boldsymbol{E}_{\boldsymbol{\infty}}$**, -10%** | **Average *T_2_^*^* (ms)** |
| **Joint Forces (N)** | 1 | 0.94 | 0.89 | 0.94 | 0.83 | -0.37 |
| $\boldsymbol{E}_{\boldsymbol{o}}$**, -5%** | *0.017* | 1 | 0.77 | 0.99 | 0.94 | -0.42 |
| $\boldsymbol{E}_{\boldsymbol{\infty}}$**, -5%** | *0.033* | *0.103* | 1 | 0.77 | 0.71 | -0.14 |
| $\boldsymbol{E}_{\boldsymbol{o}}$**, -10%** | *0.017* | *0.003* | *0.103* | 1 | 0.94 | -0.43 |
| $\boldsymbol{E}_{\boldsymbol{\infty}}$**, -10%** | *0.058* | *0.017* | *0.136* | *0.017* | *1* | -0.2 |
| **Average *T_2_^*^* (ms)** | *0.497* | *0.419* | *0.803* | *0.419* | *0.713* | 1 |
|  |  |  |  |  |  |  |
| **Patellar Tendon** | | | | | | |
| **ρ**  **p** | **Joint Forces (N)** | $\boldsymbol{E}_{\boldsymbol{o}}$**, -5%** | $\boldsymbol{E}_{\boldsymbol{\infty}}$**, -5%** | $\boldsymbol{E}_{\boldsymbol{o}}$**, -10%** | $\boldsymbol{E}_{\boldsymbol{\infty}}$**, -10%** | **Average *T_2_^*^* (ms)** |
| **Joint Forces (N)** | 1 | 0.89 | 0.77 | 0.54 | 0.77 | 0.09 |
| $\boldsymbol{E}_{\boldsymbol{o}}$**, -5%** | *0.033* | 1 | 0.94 | 0.83 | 0.94 | 0.42 |
| $\boldsymbol{E}_{\boldsymbol{\infty}}$**, -5%** | *0.103* | *0.017* | 1 | 0.94 | 0.99 | 0.31 |
| $\boldsymbol{E}_{\boldsymbol{o}}$**, -10%** | *0.297* | *0.058* | *0.017* | 1 | 0.94 | 0.37 |
| $\boldsymbol{E}_{\boldsymbol{\infty}}$**, -10%** | *0.103* | *0.017* | *0.003* | *0.017* | 1 | 0.31 |
| **Average *T_2_^*^* (ms)** | *0.919* | *0.419* | *0.564* | *0.497* | *0.563* | 1 |
|  |  |  |  |  |  |  |
| **Lateral Femoral Condylar Cartilage** | | | | | | |
| **ρ**  **p** | **Joint Forces (N)** | $\boldsymbol{E}_{\boldsymbol{o}}$**, -5%** | $\boldsymbol{E}_{\boldsymbol{\infty}}$**, -5%** | $\boldsymbol{E}_{\boldsymbol{o}}$**, -10%** | $\boldsymbol{E}_{\boldsymbol{\infty}}$**, -10%** | **Average *T_2_^*^* (ms)** |
| **Joint Forces (N)** | 1 | 0.94 | 0.09 | 0.14 | 0.03 | 0.37 |
| $\boldsymbol{E}_{\boldsymbol{o}}$**, -5%** | *0.017* | 1 | -0.03 | 0.26 | 0.09 | 0.26 |
| $\boldsymbol{E}_{\boldsymbol{\infty}}$**, -5%** | *0.919* | *0.99* | 1 | 0.43 | 0.66 | 0.94 |
| $\boldsymbol{E}_{\boldsymbol{o}}$**, -10%** | *0.803* | *0.658* | *0.419* | 1 | 0.94 | 0.54 |
| $\boldsymbol{E}_{\boldsymbol{\infty}}$**, -10%** | *0.99* | *0.919* | *0.175* | *0.017* | 1 | 0.71 |
| **Average *T_2_^*^* (ms)** | *0.497* | *0.658* | *0.017* | *0.297* | *0.136* | 1 |
|  |  |  |  |  |  |  |
| **Medial Femoral Condylar Cartilage** | | | | | | |
| **ρ**  **p** | **Joint Forces (N)** | $\boldsymbol{E}_{\boldsymbol{o}}$**, -5%** | $\boldsymbol{E}_{\boldsymbol{\infty}}$**, -5%** | $\boldsymbol{E}_{\boldsymbol{o}}$**, -10%** | $\boldsymbol{E}_{\boldsymbol{\infty}}$**, -10%** | **Average *T_2_^*^* (ms)** |
| **Joint Forces (N)** | 1 | 0.26 | -0.09 | 0.26 | 0.43 | -0.26 |
| $\boldsymbol{E}_{\boldsymbol{o}}$**, -5%** | *0.658* | 1 | 0.66 | 0.89 | 0.83 | 0.77 |
| $\boldsymbol{E}_{\boldsymbol{\infty}}$**, -5%** | *0.919* | *0.175* | 1 | 0.43 | 0.27 | 0.71 |
| $\boldsymbol{E}_{\boldsymbol{o}}$**, -10%** | *0.658* | *0.419* | *0.033* | 1 | 0.89 | 0.49 |
| $\boldsymbol{E}_{\boldsymbol{\infty}}$**, -10%** | *0.419* | *0.058* | *0.658* | *0.033* | 1 | 0.37 |
| **Average *T_2_^*^* (ms)** | *0.658* | *0.103* | *0.136* | *0.366* | *0.497* | 1 |
|  |  |  |  |  |  |  |
| **Lateral Meniscus** | | | | | | |
| **ρ**  **p** | **Joint Forces (N)** | $\boldsymbol{E}_{\boldsymbol{o}}$**, -5%** | $\boldsymbol{E}_{\boldsymbol{\infty}}$**, -5%** | $\boldsymbol{E}_{\boldsymbol{o}}$**, -10%** | $\boldsymbol{E}_{\boldsymbol{\infty}}$**, -10%** | **Average *T_2_^*^* (ms)** |
| **Joint Forces (N)** | 1 | -0.09 | -0.37 | 0.71 | 0.03 | 0.31 |
| $\boldsymbol{E}_{\boldsymbol{o}}$**, -5%** | *0.919* | 1 | 0.49 | 0.6 | 0.77 | 0.37 |
| $\boldsymbol{E}_{\boldsymbol{\infty}}$**, -5%** | *0.497* | *0.355* | 1 | 0.14 | 0.6 | 0.2 |
| $\boldsymbol{E}_{\boldsymbol{o}}$**, -10%** | *0.136* | *0.241* | *0.803* | 1 | 0.49 | 0.49 |
| $\boldsymbol{E}_{\boldsymbol{\infty}}$**, -10%** | *0.99* | *0.103* | *0.241* | *0.356* | 1 | 0.6 |
| **Average *T_2_^*^* (ms)** | *0.563* | *0.497* | *0.713* | *0.355* | *0.242* | 1 |
|  |  |  |  |  |  |  |
| **Medial Meniscus** | | | | | | |
| **ρ**  **p** | **Joint Forces (N)** | $\boldsymbol{E}_{\boldsymbol{o}}$**, -5%** | $\boldsymbol{E}_{\boldsymbol{\infty}}$**, -5%** | $\boldsymbol{E}_{\boldsymbol{o}}$**, -10%** | $\boldsymbol{E}_{\boldsymbol{\infty}}$**, -10%** | **Average *T_2_^*^* (ms)** |
| **Joint Forces (N)** | 1 | -0.43 | -0.2 | -0.43 | -0.09 | -0.14 |
| $\boldsymbol{E}_{\boldsymbol{o}}$**, -5%** | *0.419* | 1 | 0.71 | 0.89 | 0.54 | 0.37 |
| $\boldsymbol{E}_{\boldsymbol{\infty}}$**, -5%** | *0.713* | *0.136* | 1 | 0.94 | 0.89 | 0.83 |
| $\boldsymbol{E}_{\boldsymbol{o}}$**, -10%** | *0.419* | *0.033* | *0.017* | 1 | 0.77 | 0.71 |
| $\boldsymbol{E}_{\boldsymbol{\infty}}$**, -10%** | *0.919* | *0.297* | *0.03* | *0.103* | 1 | 0.6 |
| **Average *T_2_^*^* (ms)** | *0.803* | *0.497* | *0.058* | *0.136* | *0.241* | 1 |
