# Supplementary material for: Multiscale correlations between joint and tissue-specific biomechanics and anatomy in postmortem ovine stifles
Source: Sci Rep. 2025 Feb 7;15:4630. doi: 10.1038/s41598-025-87491-w (PMC11806062; doi:10.1038/s41598-025-87491-w)
Supplement: Supplementary file 6 — Supplementary Material 6 [file 41598_2025_87491_MOESM6_ESM.docx]

**Supplemental Table 4. Relaxation time constants** $\boldsymbol{\tau}_{\boldsymbol{min}}$ **and** $\boldsymbol{\tau}_{\boldsymbol{max}}$ **values in seconds (s) for the menisci, cartilage (from either medial and lateral femoral condyles),** **ligaments (ACL, PCL, MCL and LCL) and patellar tendon for the tested ovine specimens (total six – one stifle per each animal) measured under 5 and 10% strain** **the shorter time constant is referred to as** $\boldsymbol{\tau}_{\boldsymbol{min}}$ **and the longer one as** $\boldsymbol{\tau}_{\boldsymbol{max}}$**. The sex of each specimen is also provided, M- Male and F-Female.**

**Tissue-specific values of** $\boldsymbol{\tau}_{\boldsymbol{min}}$ **(shorter time constant) measured under 5% strain**

| **Specimen** | **Lateral Meniscus** | **Medial Meniscus** | **Cartilage (LFC)** | **Cartilage (MFC)** | **ACL** | **MCL** | **PCL** | **LCL** | **Patellar Tendon** |
| --- | --- | --- | --- | --- | --- | --- | --- | --- | --- |
| **S-1 (F)** | 13.07 | 28.39 | 64.84 | 40.56 | 6.15 | 31.34 | 44.07 | 27.48 | 41.61 |
| **S-2 (M)** | 24.84 | 12.05 | 25.12 | 29.42 | 48.45 | 30.64 | 25.11 | 21.76 | 34.39 |
| **S-3 (F)** | 19.03 | 28.8 | 38.4 | 13.76 | 20.58 | 16.78 | 45.19 | 21.13 | 32.37 |
| **S-4 (F)** | 19.44 | 38.17 | 27.66 | 30.88 | 33.77 | 25.57 | 47.92 | 37.71 | 21.78 |
| **S-5 (M)** | 43.05 | 20.28 | 13.15 | 12.65 | 31.42 | 38.21 | 27.1 | 38.21 | 28.63 |
| **S-6 (F)** | 10.93 | 21.64 | 42.35 | 11.44 | 21.58 | 22.85 | 29.68 | 31.71 | 22.23 |

**Tissue-specific values of** $\boldsymbol{\tau}_{\boldsymbol{m}\boldsymbol{ax}}$ **(longer time constant) measured under 5% strain**

| **Specimen** | **Lateral Meniscus** | **Medial Meniscus** | **Cartilage (LFC)** | **Cartilage (MFC)** | **ACL** | **MCL** | **PCL** | **LCL** | **Patellar Tendon** |
| --- | --- | --- | --- | --- | --- | --- | --- | --- | --- |
| **S-1 (F)** | 190.98 | 262.95 | 416.17 | 517.33 | 348.85 | 523.81 | 435.89 | 343.42 | 628.23 |
| **S-2 (M)** | 298.48 | 102.77 | 538.41 | 363.74 | 720.74 | 480.27 | 441.71 | 318.44 | 483.08 |
| **S-3 (F)** | 201.19 | 311.8 | 766.68 | 174.84 | 330.12 | 521.02 | 431.58 | 387 | 490.53 |
| **S-4 (F)** | 137.16 | 296 | 464.18 | 352.94 | 350.54 | 402.95 | 427.13 | 403.52 | 256.02 |
| **S-5 (M)** | 416.54 | 235.86 | 127.82 | 132.44 | 443.55 | 516.66 | 398.2 | 516.66 | 460.43 |
| **S-6 (F)** | 162.66 | 217.7 | 554.07 | 236.48 | 272.48 | 304.8 | 391.11 | 450.06 | 329.14 |

**Tissue-specific values of** $\boldsymbol{\tau}_{\boldsymbol{m}\boldsymbol{in}}$ **(shorter time constant) measured under 10% load**

| **Specimen** | **Lateral Meniscus** | **Medial Meniscus** | **Cartilage (LFC)** | **Cartilage (MFC)** | **ACL** | **MCL** | **PCL** | **LCL** | **Patellar Tendon** |
| --- | --- | --- | --- | --- | --- | --- | --- | --- | --- |
| **S-1 (F)** | 13.6 | 26.01 | 44.82 | 37.84 | 22.01 | 17.9 | 6.79 | 15.97 | 20.11 |
| **S-2 (M)** | 17.96 | 22.88 | 40.31 | 28.21 | 16.08 | 16.15 | 12.02 | 17.96 | 12.88 |
| **S-3 (F)** | 30.06 | 34.44 | 19.89 | 12.97 | 17.74 | 20.24 | 15.86 | 10.18 | 13.75 |
| **S-4 (F)** | 43.42 | 51.19 | 46.44 | 24.55 | 22.75 | 9.26 | 12.34 | 25.06 | 22.07 |
| **S-5 (M)** | 48.94 | 27.79 | 40.83 | 48.35 | 22.94 | 7.44 | 22.6 | 7.54 | 34.72 |
| **S-6 (F)** | 24.38 | 35.15 | 16.55 | 13.3 | 17.95 | 15.72 | 7.34 | 17.05 | 15.35 |

**Tissue-specific values of** $\boldsymbol{\tau}_{\boldsymbol{m}\boldsymbol{ax}}$ **(longer time constant) measured under 10% load**

| **Specimen** | **Lateral Meniscus** | **Medial Meniscus** | **Cartilage (LFC)** | **Cartilage (MFC)** | **ACL** | **MCL** | **PCL** | **LCL** | **Patellar Tendon** |
| --- | --- | --- | --- | --- | --- | --- | --- | --- | --- |
| **S-1 (F)** | 173.04 | 234.77 | 315.17 | 1000 | 349.06 | 428.77 | 245.07 | 553.98 | 443.07 |
| **S-2 (M)** | 246.02 | 180.7 | 341.59 | 285.82 | 622.38 | 434.83 | 342.31 | 431.72 | 366.35 |
| **S-3 (F)** | 270.49 | 291.55 | 249 | 154.36 | 455.8 | 399.22 | 345.45 | 354.67 | 371.32 |
| **S-4 (F)** | 299.66 | 398.28 | 511.97 | 234.9 | 429.13 | 388.24 | 355.51 | 470.04 | 397.18 |
| **S-5 (M)** | 448.69 | 239.77 | 533.75 | 357.4 | 520.79 | 238.27 | 455.76 | 241.47 | 548.81 |
| **S-6 (F)** | 213.66 | 245.99 | 409.21 | 249.64 | 354.88 | 393.87 | 428.33 | 392.99 | 364.14 |
